# Complete Genome Sequence of Lactiplantibacillus plantarum Strain VHProbi V38, Isolated from Kimchi Soup

**DOI:** 10.1128/mra.00493-22

**Published:** 2022-10-03

**Authors:** Hongchang Cui, Jingyan Zhang, Zhi Duan

**Affiliations:** a Qingdao Vland Biotech Inc., Qingdao, China; University of Maryland School of Medicine

## Abstract

Lactiplantibacillus plantarum strain VHProbi V38 is a traditional lactic acid bacterial probiotic that has been commercialized for several years. Here, we report the whole-genome sequence of this strain. Sequencing yielded a 3,197,704-bp genome and 3,050 protein-coding genes.

## ANNOUNCEMENT

Samples (1 mL) of kimchi soup purchased from the supermarket were inoculated onto de Man-Rogosa-Sharpe agar (Haibo, China) plates and then incubated under anaerobic conditions at 37°C for 24 h to enrich bacterial strains. A bacterial strain was picked up and designated V38. 16S rRNA sequencing studies according to the method described by Liu showed that the isolate belongs to the species Lactiplantibacillus plantarum (1). A combined sequencing strategy using both Illumina HiSeq 2500 and PacBio RS II technologies was used to sequence the genome of this strain. The strain was cultured for DNA extraction under the same conditions as for strain isolation. Total DNA was extracted using a genomic DNA purification kit (Promega, USA) following the manufacturer’s instructions. The Illumina sequencing library was prepared from the sheared DNA fragments using the NEXTflex rapid DNA-seq kit (Bioo Scientific, USA) and sequenced in Illumina paired-end format (2 × 150 bp). For PacBio sequencing, DNA spun in a g-TUBE (Covaris, MA) was sheared into ~10-kb fragments. The DNA fragments were then purified, end repaired, and ligated with SMRTbell sequencing adapters (PacBio, CA) following the manufacturer’s instructions and sequenced on a single-molecule real-time (SMRT) cell using standard methods. Finally, 6,812,090 raw reads and 6,718,688 clean reads were generated with a coverage depth of 328.01-fold using the Illumina sequencing platform. Using the PacBio RS II sequencing platform, 138,320 raw reads (*N*_50_, 7,572 bp) were generated. The Illumina-generated sequences were quality filtered using Sickle v1.33 (https://github.com/najoshi/sickle), and low-quality sequences were removed before assembly (2). The PacBio raw reads were assembled into a scaffold using Unicycler v0.4.8 (3). Pilon v1.22 was used for error correction of the PacBio assembly results (4). If there was overlap of a certain length at both ends of the assembly sequence, the sequence was looped and one end of the overlapping sequence cut off to obtain complete genome and plasmid sequences. Glimmer v3.02 (5), tRNAscan-SE v2.0 (6), and Barrnap v0.9 (https://github.com/tseemann/barrnap/) were used to predict protein-coding genes, tRNAs, and rRNAs, respectively. Genome annotation was conducted using the NCBI Prokaryotic Genome Annotation Pipeline (PGAP) v6.1 (7). Default parameters were used for all software unless otherwise specified.

The complete genome sequence of *L. plantarum* VHProbi V38 contains a circular 3,197,704-bp chromosome with a GC content of 44.6%. There are 3,050 coding genes, 16 rRNA genes (5S, 16S, and 23S rRNA genes), 70 tRNA genes, 4 noncoding RNA (ncRNA) genes, and 52 pseudogenes in the genome. There are also 8 plasmids (Table 1).

**TABLE 1:** Summary data of the plasmids

Name	Size (bp)	Type	No. of genes	GenBank protein accession no. for replicon	GenBank accession no.
pAV38	71,116	Circular	71		CP092502.1
pBV38	35,348	Circular	34		CP092503.1
pCV38	13,968	Circular	14	UQK35762	CP092504.1
pDV38	9,036	Circular	12		CP092505.1
pEV38	8,624	Circular	8	UQK35777	CP092506.1
pFV38	8,520	Circular	13	UQK35788	CP092507.1
pGV38	6,858	Circular	9	UQK35799	CP092508.1
pHV38	3,495	Circular	5		CP092509.1

### Data availability.

The 16S rRNA sequence and complete genome sequence of *L. plantarum* VHProbi V38 have been deposited at GenBank under accession numbers OP077205 and CP092501 to CP092509, SRA accession numbers SRR19160253 and SRR19160254, BioProject accession number PRJNA824600, and BioSample accession number SAMN26145752.
